# Aquaporin Activity to Improve Crop Drought Tolerance

**DOI:** 10.3390/cells7090123

**Published:** 2018-08-29

**Authors:** Avat Shekoofa, Thomas R. Sinclair

**Affiliations:** 1 Plant Sciences Department, University of Tennessee, West TN Research & Education Center, Jackson, TN 38301-3201, USA; 2 Crop and Soil Sciences Department, North Carolina State University, Raleigh, NC 27695-7620, USA; trsincla@ncsu.edu

**Keywords:** aquaporins (AQPs), water deficit stress, high vapor pressure deficit (VPD), limited-transpiration (TR_lim_) trait

## Abstract

In plants, aquaporins (AQP) occur in multiple isoforms in both plasmalemma and tonoplast membranes resulting in regulation of water flow in and out of cells, and ultimately, water transfer through a series of cells in leaves and roots. Consequently, it is not surprising that physiological and molecular studies have identified AQPs as playing key roles in regulating hydraulic conductance in roots and leaves. As a result, the activity of AQPs influences a range of physiological processes including phloem loading, xylem water exit, stomatal aperture and gas exchange. The influence of AQPs on hydraulic conductance in plants is particularly important in regulating plant transpiration rate, particularly under conditions of developing soil water-deficit stress and elevated atmospheric vapor pressure deficit (VPD). In this review, we examine the impact of AQP activity and hydraulic conductance on crop water use and the identification of genotypes that express soil water conservation as a result of these traits. An important outcome of this research has been the identification and commercialization of cultivars of peanut (*Arachis hypogaea* L.), maize (*Zea mays* L.), and soybean (*Glycine max* (Merr) L.) for dry land production systems.

## 1. Introduction

Aquaporins (AQPs) play critical roles in controlling water transfer into and out of plant cells, which is crucial in the transport of water in and around plants, and in maintaining cell viability. The activity of plant AQPs appears to be intimately linked to expressions of plant tolerance to biotic and abiotic stresses [1]. Drought, in particular, is often the most limiting abiotic stress causing crop yield losses because nearly all crops suffer from at least short periods of drought sometime during their growing cycle [2]. Total annual crop damage from drought in the United States has been estimated at $6 to $8 billion [3].

The opening and closing of the AQP gates [4] result in the control of water transport across cell membranes and through a series of cells. AQPs are present both in the plasma membrane of plant cells and in tonoplast membranes that enclose vacuoles, which often account for the majority of plant cell volume. Consequently, AQPs play important roles in the overall determination of both plant water balance, including osmoregulation, single-cell expansion, and long-distance transport [5], and effective plant water use [6,7,8].

As a result of AQP sensitivity to variables such as temperature and cell water status, plant overall hydraulic conductance and water status are responsive to changes in their environment [8] including drought and varying atmospheric vapor pressure deficit (VPD). For example, Sakuarai-Ishikawa et al. [9] showed a close coupling between diurnal changes in rice (*Oryza sativa* L.) transpiration rate and root conductance. Their conclusion was that conductance was responsive to transpiration rate. The transpiration rate demand triggered an increase in gene expression related to AQPs activity.

## 2. Plant Water Use

Crop growth is intimately linked to transpiration and little can be done to alter this relationship [10]. The timing in the use of the available water can, however, be critical in determining the yield outcome in differing approaches to water use [11]. Aggressive water use with maintenance of open stomata allows immediate sustained growth but requires timely rainfall or irrigation to avoid the rapid development of severe water-deficit stress. In contrast, conservative approaches in the timing of water use avoid severe stress, but the crop may suffer from lost productivity during periods of partial stomatal closure when transpiration rate is restricted. On balance, simulation analyses over many growing seasons have clearly shown a major yield advantage in most cases resulting from the conservative approach [12,13]. This outcome was obtained due to the benefit of having adequate water at the end of the growing season to sustain physiological activity and complete grain production.

Identification of specific plant traits associated with AQP activity needs to be done in the context of the temporal water use over the entire growing season. For example, traits that might be expected to improve plant survival of a severe drought period likely do not offer a useful approach for season-long use of available water by annual crops. This results because survival of annual crops during a period of low water availability still means economically devastating yields for the grower due to the overall lack of water to support crop growth. Also, misunderstandings in the seasonal dynamics of water use extend to attempts to relate AQP expression based on one-time, short duration (hours) studies in extrapolating AQP activity to the seasonal dynamics of water use by crops in the field. For example, a change in the level of an AQP in response to an osmotic stress [14] cannot be used to predict the overall yield response to the temporally dynamic changes in water-deficit in the field.

To ameliorate the impact of drought on crop yields, the critical need is commonly to improve crop performance in expectation of developing soil water deficits. The traits that appear to be particularly desirable to improve yield performance under water-deficit conditions generally are associated with limiting water use by the crop early in the growing season so that water is conserved for use later in season to sustain plant productivity during reproductive development [15,16,17,18]. To achieve soil water conservation early in the growing season there are two prime mechanisms for water conservation, both of which appear to be closely related to AQPs activity: (i) transpiration sensitivity to soil drying and (ii) transpiration sensitivity to high atmospheric vapor pressure deficit (VPD). The functioning of these mechanisms seems to be directly related to AQPs-regulated hydraulic conductance in plant tissue. The consequence of both mechanisms invokes decreasing gas exchange and protection of the hydraulic system of the plant to prevent severe plant dehydration [8].

## 3. Hydraulic Conductance 

Hydraulic conductance impacts on water flux to and in plant leaves is a critical variable determining the water supply to support leaf transpiration rate (TR) [19,20]. Stomatal conductance can adjust to assure that TR matches the water supply so that leaf desiccation is avoided. Under conditions of limited water supply during drought, partial stomatal closure occurs to balance TR with water flux into the leaves [21].

Considerable research has been done on changes in plant hydraulic conductance under water-deficit conditions. Much of this research, however, has been focused on extreme drought with perennial species. In these cases, embolism formation in xylem vessels occurs when plant water potential decreases to about −1.0 MPa or less [22,23]. In terms of improving annual crop yields under water deficit, however, such severe stress levels are not usually relevant.

For annual crop species, decreases in hydraulic conductance in early stages of soil drying associated with AQP activity, including situations of decrease even before transpiration rate is decreased, are particularly important [24,25]. These decreases in hydraulic conductance have been reported to occur in roots [24,26], leaves [27,28], or both [29] in crop plants. As discussed later, AQP inhibitors have been used to identify differences among genotypes in crop species to identify lines with AQP expression linked to desired hydraulic conductance behavior, and potentially increased drought tolerance.

## 4. Soil Drying

AQPs are likely to have impacts on crop physiological performance under drought conditions through their effects on water transport, and ultimately stomatal conductance [8]. A decrease in plant hydraulic conductance associated with early stages of soil drying allows the possibility of partial closure of stomata early in the soil drying cycle. The benefit of such behavior for annual row crops is that soil water conservation is initiated earlier in the soil drying cycle, resulting in decreased rate in the use of water and allowing for sustained physiological activity as the water deficit and growing season progresses. In fact, initiation of partial stomatal closure has now been identified in individual genotypes in nearly all major crop species [10].

**Figure 1:**
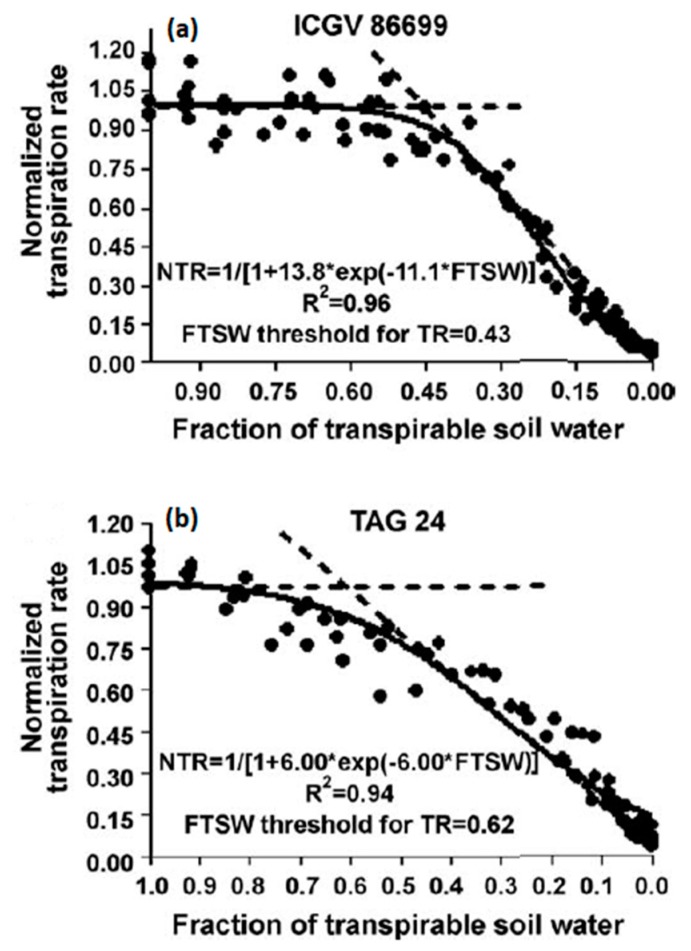
Graph of normalized transpiration rate relative to the transpiration rate of well-watered plants for each genotype plotted against fraction of transpirable soil water for 6 week old peanut plants grown on mineral soil in 2.4 L pots [32]. These plants were subjected to controlled soil drying over about 2 weeks. The normalized was done to allow convenient comparison between genotypes ICGV 8699 (**a**) and TAG 24 (**b**).

One of the more successful efforts in identification of a genotype with the early partial stomatal closure with soil drying was in peanut (*Arachis hypogaea* L.) [30]. Nearly all peanut lines, like in most species, exhibit a decrease in normalized TR in response to soil drying when there is only about 0.3 to 0.4 fraction of transpiration soil water (FTSW) remaining in the soil. This threshold for the decrease in transpiration rate matches the soil water content when hydraulic conductance in the soil decreases to where it limits water transport to the root [31]. The nearly universal response of TR to soil drying is illustrated in Figure 1 by ICGV 8699. However, a few peanut lines were found that initiate partial stomata closure at FTSW > 0.6 as shown by TAG 24 in Figure 1 [32]. The supposition was that the TAG 24 line developed an especially low plant hydraulic conductance and that when coupled with low soil hydraulic conductance resulted in decreased transpiration rate at higher soil water content than usually observed.

Within U.S. virginia-type peanut, the breeding line N12006ol was found to begin stomatal closure at a high threshold and was found to be associated with higher yield under drought conditions. This line is now in the process of commercial release [30].

In rice (*Oryza sativa* L.), Nada and Abogadallah [33] showed significant difference between indica and japonica rice cultivars for the threshold for decrease in FTSW with water-deficit stress under a field condition. Measurements of root and leaf hydraulic conductivity indicated that stomatal conductance and transpiration rates were more related to root rather than leaf hydraulic conductivity. This study showed that roots have lower water permeability than leaves under drought. Furthermore, they found that the differences in AQP expression profiles could account for the significantly higher rates of transpiration in the indica rice at least at a certain point during the day (i.e., 9:00 and 13:00 in drought stressed rice, but not at 17:00) compared to japonica cultivars. More importantly, there was a strong induction of aquaporins by drought in the leaves at 9:00 a.m. in all cultivars that was stronger in indica rice than japonica rice. The diminished expression of PIP2;1, PIP2;2, andPIP2;4, which have high water transport activity in roots although they are expressed in leaves to considerably higher levels, would further intensify the shortage of water supply to leaves at 9:00 (or later during the day) and cause rapid decline in plant water budget resulting in stomatal depression at midday [33].

Moreover, Sakurai et al. [9] reported that in rice aquaporins, such as OsPIP2;1 and OsPIP2;2, which are localized both in leaf blades and in roots, may play a constitutive role in water transport in both organs, irrespective of the diurnal cycle and stress conditions.

## 5. High Evaporative Demand [or High Vapor Pressure Deficit (VPD)]

Under high evaporative demand, transcellular AQP-controlled water flow may become a significant determinant of the leaf water balance and stomatal conductance [34]. In achieving this balance, it is necessary under high VPD conditions to have partial stomatal closure so that TR is limited to a rate that matches water flow to the stomata. This response is related to hydraulic conductance limitations associated with AQPs activity, although no consistent correspondence has been found between hydraulic conductance and abundance of specific AQPs [19].

The overall sensitivity to VPD has been referred to a limited-transpiration (TR_lim_) trait. This response was first fully documented in the soybean (*Glycine max* (Merr) L.) genotype PI 416937 [35]. The TR_lim_ was found to occur when VPD increased to about 2.1 kPa. Sinclair et al. [25] showed that the TR_lim_ response in PI 416937 was associated with a low hydraulic conductance in the leaves between the leaf xylem and into the guard cells.

**Figure 2:**
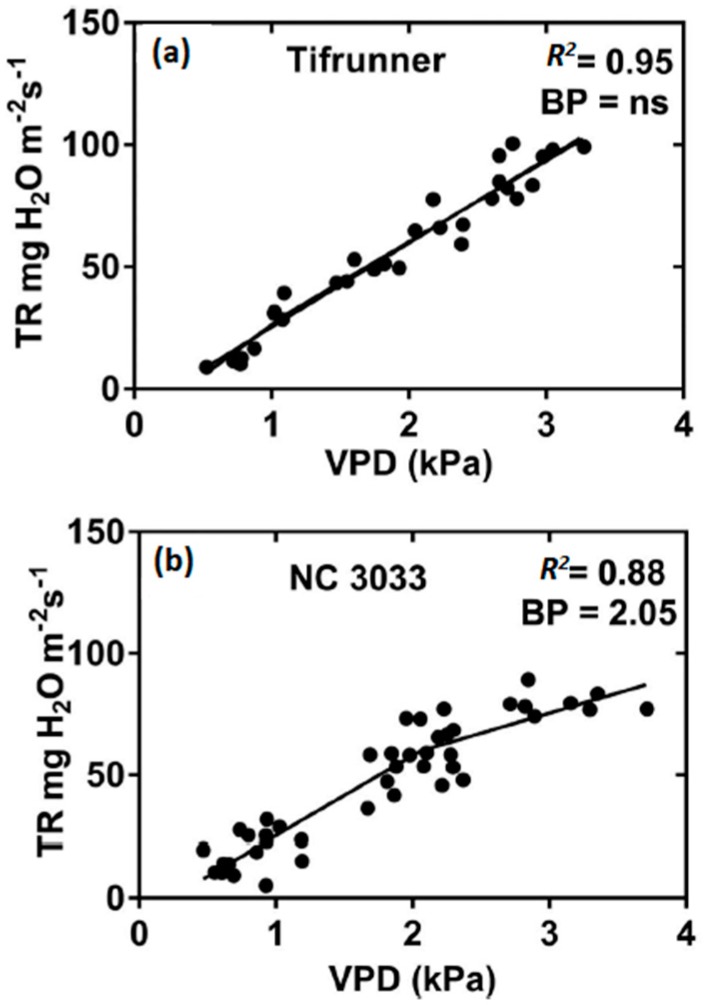
Plot of transpiration rate (TR) vs. vapor pressure deficit (VPD) of 5 week old plants of (**a**) Tifrunner and (**b**) NC 3033 [37]. Entire plants were enclosed in individual mini-chambers (21 L) and TR was measured gravimetric for 1-exposure at each VPD. Regression analysis showed a linear increase in TR for Tifrunner over the entire range of tested VPD while a two-segment response was found for NC 3033 with a breakpoint (BP) between segments at 2.05 kPa.

Expression of TR_lim_ has now been documented in selected genotypes of several crop species including peanut, soybean (*Glycine max* (L.) Merr.), maize (*Zea mays* L.), sorghum (*Sorghum bicolor* L.), pearl millet (*Pennisetum glaucum* L.), and chickpea (*Cicer arietinum* L.) [36]. Recently, the TR_lim_ trait was identified in cotton (*Gossypium hirsutum* L.) with expression of a limitation at 1.90 kPa (Shekoofa et al., 2018 unpublished data). Also, genotypic variation in TR response to different VPD was identified in breeding, commercial and recombinant inbred lines (RILs) within crop species as illustrated in Figure 2 in the comparison of two peanut cultivars [37].

Since direct, experimental measurement of the TR response at high VPD is laborious and only a few genotypes can be characterized at one time, it was useful to develop an initial screen for the TR_lim_ trait under high VPD to narrow the number of lines to be directly tested for the trait. One approach was to explore the possibility that an AQP inhibitor might relate to the transpiration response to high VPD. Elemental metals have been used as AQP inhibitors. One of the most potent and commonly used is mercury [38]. In addition, silver and gold are AQP inhibitors [39]. Transpiration rate has also been found to be inhibited by zinc treatment [40,41].

One of the first studies with AQP inhibitors was feeding of de-rooted shoots of four soybean genotypes with mercury and silver [39]. While all four genotypes were sensitive to mercury, PI 416937 proved to be insensitive to exposure to silver in contrast to the other three genotypes. The hypothesis for the insensitivity of PI 416937 to silver was that the silver-sensitive isoforms of AQPs were not active in this particular genotype. The lack of activity of an isoform AQP was hypothesized to result in a lower leaf hydraulic conductance, and hence, the observed decrease in transpiration rate at low VPD [39]. Further, it was found that there was up-regulation of AQP PIP 1;7 in its roots following a silver treatment [42]. Genotype PI 416937 has been now been used as a parent to develop drought-tolerant, commercial cultivars [18].

**Figure 3:**
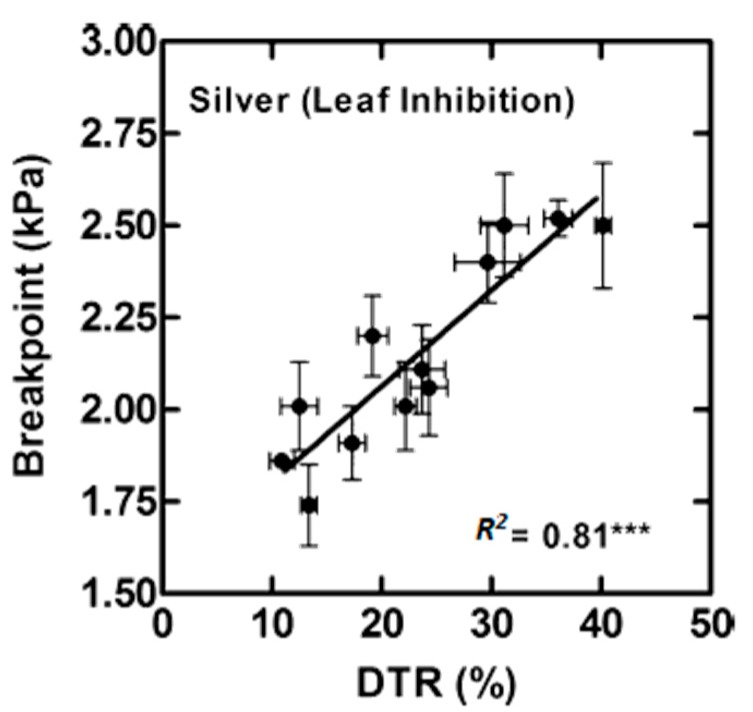
Twelve maize hybrids that were phenotyped for expressing a TR_lim_ breakpoint in response to increasing VPD were also measured for decrease in transpiration rate (DTR) when exposed to silver ions (500 μM) [43]. These results showed a high correlation the breakpoint and DTR reflecting apparent variation in aquaporins (AQP) sensitivity to silver. *** a significant correlation at *P* < 0.001.

Genotypes expressing TR_lim_ with increasing atmospheric VPD were also found in species other than soybean to have low hydraulic conductivity in their leaves. In maize, a possible correlation between the expression of the TR_lim_ trait and response to silver treatment was explored [43]. The study was done with DuPont Pioneer’s AQUAmax hybrids (The DuPont Pioneer Innovation Center, Johnston, IA, USA), which have been previously shown to express the TR_lim_ trait with increasing VPD [44]. The VPD at which TR_lim_ was expressed was found to correlate with the degree of decrease in transpiration rate when leaves were fed with silver ion (Figure 3, [43]). The mean yield advantage across a wide range of field environments for AQUAmax hybrids as compared to non-AQUAmax hybrids under rainfed conditions was 36, 53, and 22 g m^−2^, respectively [18].

The use of silver for the screening of genotypes for the trait of TR_lim_ under high VPD has been proposed in other species including peanut and sorghum [45,46]. Shekoofa et al. [47] studied the expression of the TR_lim_ trait in two peanut progeny populations: runner and virginia type. Two levels of screening were performed on both populations. Initially the populations were screened by feeding silver ions (200 μM) to a large number of genotypes in each population. Then, a smaller number of recombinant inbred lines (RILs) were selected for direct measure of TR response to VPD. The study showed that an effective expression of the TR_lim_ trait occurred in about 25% of the progeny in each of the two populations supporting breeding for transfer of the TR_lim_ trait to progeny genotypes [47].

As previously indicated [19,20], plants hydraulic conductance behavior is regulated partially by AQPs activity, however no consistent correspondence has been found between hydraulic conductance and abundance of specific AQPs [19]. For example, it has been difficult to show a common response of root PIP AQP expression and PIP protein abundance under drought conditions [19]. Among the 37 PIP genes studied, 15 were down-regulated, 13 upregulated, and nine unaltered. Even in the same experiment some PIP genes were down-regulated, others up-regulated, and others unaltered.

Pou et al. [20] indicated that the expression patterns in grapevine (*Vitis vinifera* L. cv Chardonnay) reveal that the contribution to flow from AQPs may be relatively complex and highly regulated, probably involving different isoforms at different stages of water stress and recovery.

## 6. Molecular Observations

**Figure 4:**
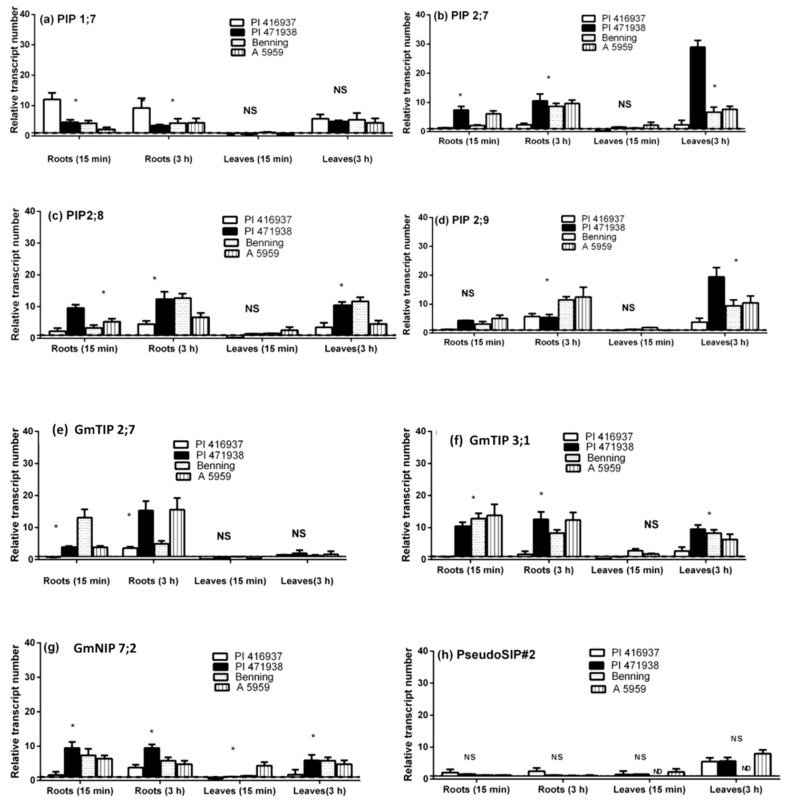
RNA abundance response relative to untreated tissues 15 min and 180 min after treatment with 200 μM AQPs inhibitor [silver nitrate (AgNO3)] of eight AQPs: (**a**) PIP 1;7; (**b**) PIP 2;7; (**c**) PIP 2;8; (**d**) PIP 2;9; (**e**) GmTIP 2;7; (**f**) GmTIP 3;1; (**g**) GmNIP 7;2; and (**h**) PesudoSIP#2 in soybean roots and leaves [42]. Bars indicate transcript abundance ±S.E. Those noted with * are significantly different at *P* < 0.05, NS are non-significant and ND are not detectable.

Soybean genotype PI 416937 has previously been found to have low leaf hydraulic conductivity, which was hypothesized to be the basis for limited transpiration (TR_lim_) rates under high vapor pressure deficit [39]. Devi et al. [42] measured RNA abundance of eight AQPs for soybean lines expressing the TR_lim_ (PI 416937, delayed wilting) and not expressing the TR_lim_. After treatment with the AQP inhibitors, there were observed differences in the abundance of RNA among genotypes [42]. All measured RNA was either unchanged or up-regulated in response to treatment of the intact plants with silver as compared to untreated plants (Figure 4, [42]). Up-regulation of almost all AQPs was observed across the four genotypes in roots as quickly as 15 min after initiation of silver treatments, except for PSEUDOSIP#2 (Figure 4h, [42]). In leaves, there was no or very little change in transcript abundance, 15 min after initiation of silver treatments.

Different responses of AQPs to water-deficit stress were found in upland (drought-resistant) and lowland (drought-sensitive) rice [14]. PIP proteins increase markedly in the roots of both types, but only in upland rice leaves. OsPIP1;2, OsPIP1;3, OsPIP2;1, and OsPIP2;5 mRNA levels in roots and OsPIP1;2 and OsPIP1;3 mRNA levels in leaves were significantly up-regulated in upland rice, but their expression was unchanged or down-regulated in lowland rice, indicating that AQPs present in the same species, but in different cultivars, can respond differently to drought depending on their overall tolerance to water deficits [14].

Nada and Abogadallah [33] concluded that midday reduction in photosynthesis in rice is mainly due to unbalanced expression of AQPs in leaves and roots resulting in rapid depletion of leaf water and subsequent inhibition of photosynthesis. Therefore, the more negative water status in rice and in particular indica cultivars, with greater drop in relative water content could be attributed to the imbalanced water transport in leaves and roots. These results indicate a strong induction of most AQPs in leaves but not in roots and hence inadequate water uptake by roots.

In sunflower (*Helianthus annuus* L.), Nardini and Salleo [48] showed that application of mercury reduced leaf hydraulic conductance. Thus, indicating that mercury-sensitive water transport proteins had been inhibited and that, consequently, putative water channels may contribute to modulating hydraulic conductance. It is also known that up-regulation or new expression of AQPs can decrease leaf hydraulic resistance of sunflower during the day by about 30%.

Pou et al. [20] reported the role of AQPs in regulating leaf hydraulic conductance in grapevine by studying effects of AQP inhibitors and AQP gene expression during drought stress and recovery. Considering both the degree of inhibition by mercury and the peaks in expression of AQPs, one set of AQPs may dominate in the leaf during the early onset of water stress (VvPIP2;2, VvTIP1;1). The expression patterns reveal that the contribution to flow from AQPs may be relatively complex and highly regulated, probably involving different isoforms at different stages of water-deficit stress and recovery.

The discrepancy between the above data from different research might arise from the different species studied, or from the different methods used to measure leaf hydraulic conductance, or/and different techniques using AQP inhibitors.

## 7. Conclusions

Despite the extensive research on AQP function in animals, in crop plants a direct linkage between AQP activity and hydraulic conductance levels that impact crop transpiration has not been resolved. Nevertheless, empirical evidence indicates that AQPs have a major role in inducing two major plant traits (i.e., transpiration sensitivity to soil drying, and transpiration sensitivity to high atmospheric vapor pressure deficit (VPD)) that allow plants to conserve soil water for sustaining plant physiological activity with developing soil water deficit. Limiting hydraulic conductance for water transport in plants as a result of AQP regulation has been associated with crop water conservation in response to early stages of soil drying and to elevated atmospheric VPD. The identification of each of these traits in the germplasm of major crop species has led to breeding efforts that have generated commercial cultivars for dryland conditions. A greater understanding of the expression of the AQP population in crop germplasm will allow for improved screening for the water conservation traits and better targeting of adapted cultivars for the range of water-deficit conditions experienced by crops.
